# Compliance with disease surveillance and notification by private health providers in South-West Nigeria

**DOI:** 10.11604/pamj.2020.35.114.21188

**Published:** 2020-04-13

**Authors:** Olusesan Ayodeji Makinde, Clifford Obby Odimegwu

**Affiliations:** 1 Demography and Population Studies Program, Schools of Public Health and Social Sciences, University of the Witwatersrand, Johannesburg, South Africa; 2 Viable Knowledge Masters, Plot C114 (Platinum Plaza), First Avenue, Gwarinpa, Abuja, Nigeria

**Keywords:** Communicable diseases, health facilities, health information system, infectious diseases, international health regulations, Nigeria, private hospitals, surveillance

## Abstract

**Introduction:**

Private health facilities are important contributors to health service delivery across several low and middle income countries. In Nigeria, they make up 33% of the health facilities, account for more than 70% of healthcare spending and over 60% of healthcare contacts are estimated to take place within them However, their level of participation in the disease surveillance system has been questioned.

**Methods:**

We conducted a cross-sectional survey of 507 private health facilities in South-West Nigeria to investigate the level of compliance with disease surveillance reporting and the factors that affect their participation.

**Results:**

We found only 40% of the private health facilities to be complying with routine disease surveillance reporting which ranged from 17% to 60% across the six states in the region. Thirty-four percent of the private health facilities had the requisite data collection tools, 49% had designated professionals assigned to health records management and only 7% of the clinicians could properly identify the three data collection tools for disease surveillance. Some important factors such as awareness of a law on disease surveillance (OR=1.55 95% CI=1.08-2.24), availability of reporting tools (OR=13.69, 95% CI=8.85-21.62), availability of a designated health records officer (OR=3.9, 95% CI=2.68-5.73), and health records officers (OR=10.51, 95%CI=2.86-67.70) and clinicians (OR=2.49, 95% CI=1.22-5.25) with knowledge of disease surveillance system were important predictive factors to compliance with disease surveillance participation.

**Conclusion:**

Private health facilities are poorly compliant with disease surveillance in Nigeria resulting in missed opportunities for prompt identification and response to threats of infectious disease outbreaks.

## Introduction

Infectious disease outbreaks arising from highly pathogenic organisms with a propensity to spread across national borders are happening more frequently. A study over a 33 year period (1980-2013) identified more than 12 000 outbreaks and found the number of outbreaks to have risen consistently across the years [1]. Between 2007 and 2014, there were three important public health emergencies of international concern: pandemic influenza H1N1 in 2009, the re-emerging wild polio virus in April 2014 and the Ebola virus disease (EVD) in August 2014 [2]. Diagnosis of rare travel-associated infections can be challenging thereby delaying appropriate definitive management. In 2009, a patient presented with uncertain symptoms at a London hospital, a few days after returning from a trip to Nigeria. Despite this history, it took about two weeks before a diagnosis of Lassa fever was reached [3]. The delay may have contributed to the demise of the patient in this instance as a result of delayed administration of a proven and effective definitive management using Ribavirin. In September 2018, two cases of Monkey pox virus were identified in the UK after two patients presented at separate health facilities for suspicious illnesses [4]. Both cases had recently been in Nigeria which had reported an ongoing outbreak of the zoonotic infection [5,6]. The World Health Assembly established the International Health Regulations (IHR) in 1969 as a framework to help control the international spread of diseases [7]. The latest review of the IHR was accomplished in 2005. The purpose and scope of the IHR (2005) are “to prevent, protect against, control and provide a public health response to the international spread of disease in ways that are commensurate with and restricted to public health risks, and which avoid unnecessary interference with international traffic and trade” [7]. The Federal Ministry of Health (FMOH) in Nigeria which is responsible for driving the health system in the country administers the national health information system [8]. The FMOH adopted the Integrated Disease Surveillance and Response (IDSR) strategy as its means of implementing the IHR in 2005 [9]. The IDSR had been put forward by the World Health Organization Regional Office for Africa as a valid means of implementing the IHR in 1998 [10]. It received great acceptance with over 90% of African countries adopting it [11]. In Nigeria, the IDSR strategy requires that health facilities in the country participate in a surveillance system that necessitates routine reporting of 41 priority diseases and conditions into the IDSR system as necessary, on a daily (epidemic prone diseases), weekly (diseases targeted for eradication or elimination) or monthly (other major diseases, events or conditions of public health importance) using the IDSR001, IDSR002 and IDSR003 forms respectively [12]. Private health facilities are important contributors to health service delivery across several low and middle income countries [13]. In Nigeria, they make up 33% of the health facilities, account for 70-80% of healthcare spending and over 60% of healthcare contacts are estimated to take place within them [14-16]. Lately, their importance in healthcare delivery has been on an upward drive arising from the incessant industrial action embarked upon by public sector workers and the perceived poor quality of service provided by public health facilities in the country [17,18]. A recent study showed that a significant proportion of deliveries take place in private health facilities in Nigeria and the level of maternal care provided by these facilities is often better than that provided by public sector facilities [19]. Also, secondary health facilities in the country which cater to a significant burden of diseases are predominantly privately owned [20]. Despite their importance in health service delivery in Nigeria, the level of participation of private health facilities in the disease surveillance system has been questioned [21]. A systematic review of the literature in 2017 which assessed the role of private health facilities in disease surveillance globally identified only one study from Nigeria [22]. The study identified had compared the functionality of a vertical tuberculosis surveillance programme between private and public health facilities and did not assess the routine disease surveillance system: the IDSR [23]. Studies that have been carried out on the performance of the disease surveillance system in the country have principally concentrated on public health facilities and public sector workers [24-27]. Thus, there is a gap in knowledge on the level of participation of private health facilities in the disease surveillance system in the country, leaving room for assumptions on their level of performance and contribution to early warning systems for epidemic prone diseases. As part of effort in addressing the knowledge gap and also as a means of identifying risks that continue to pose a threat to global health security, a study to assess the level of compliance with disease surveillance by private health facilities, determine the knowledge of private providers on disease surveillance and aimed at identifying the factors affecting their compliance with reporting in Nigeria was conducted. This study provides knowledge that is important in determining the level of compliance and the factors that affect the participation in the disease surveillance system in Nigeria, important information in addressing the shortcomings.

## Methods

We conducted a cross sectional study among 507 private health facilities within the South-West geopolitical zone (comprising of six states) of Nigeria. Ethical approval for the study was obtained from the National Health Research Ethics Committee in Nigeria (NHREC/01/01/2007-18/03/2016) and the University of the Witwatersrand Human Research Ethics Committee (Non-Medical) with Protocol Number H16/05/09. In addition, approval was sought from the commissioner of health in each state before data collection was carried out in the state. Interviewed respondents also provided written consent before taking part in the study after appropriate explanation had been provided. Data collection was done between November 2016 and November 2017. The South-West geopolitical zone of the country was selected for the study as this geopolitical zone (of the six) has the largest concentration of the private health facilities in the country (about 40%). The South-West is made of six states (Ekiti, Lagos, Ogun, Ondo, Osun and Oyo) and all states were included in the study. The Master Health Facility List published by the Federal Ministry of Health in 2014 served as the sampling frame for the study. An appropriate sample size of 424 was calculated using the Fishers formula with parameters: prevalence=50%, precision=0.05 and non-response rate=20%. This total number was then proportionally allocated to the six states based on the proportion of private health facilities each state was contributing to the aggregate in the South-West. From the sampling frame, the total number of private health facilities in each local government area (LGA) was computed for each state. This was then arranged in descending order by the number of private health facilities. The LGA with the largest number of private health facilities was subsequently selected and the data collection progressively carried out in the LGA until the sample size was achieved. In event that the sample size was not achieved in that LGA, the next LGA with the largest number of facilities was surveyed until sample size was achieved. A detailed questionnaire designed for the study was administered at the health facilities to the health records officers and clinicians (physician or nurse). The questionnaire documented information on the health facility, participation of the facility in the routine disease surveillance system, availability of data collection tools for disease surveillance, availability of a health records professional and knowledge of the health records professional and clinician (physician or nurse) on disease surveillance. The data was entered into an MS Access database designed for the study and later imported into the statistical programming environment `R´ for analysis. Frequency analysis and logistic regression analysis was done. The logistic regression investigated relationship between compliance with disease surveillance reporting by the health facility and various independent variables. Health facilities that noted that they were reporting into the disease surveillance system were coded as `1´ while those that stated that they were not reporting got `0´ in the outcomes used for the logistic regression analysis.

## Results

A total of 507 private health facilities were surveyed across the six states. Lagos had the largest contribution while Ekiti state had the lowest. Table 1 shows the distribution of health facilities surveyed by state and compliance with the disease surveillance system. Of the 507 health facilities surveyed, only 201 (40%) routinely reported notifiable diseases to the health authorities. When disaggregated by state, only Lagos (51%) and Oyo (60%) states had more than half of the health facilities surveyed reporting into the disease surveillance system. Osun, Ogun, Ondo and Ekiti states were 30%, 17%, 23% and 35% respectively (Table 1). There were no obvious differences between the reporting rates among the profit oriented and the not-for-profit oriented private health facilities (41% vs 36%). Thirty-four percent (34%) of the health facilities surveyed had the requisite data collection tools. Only 248 (49%) of the health facilities had personnel assigned to health records management. Of these 248 assigned health records officers, barely 13 (5%) could properly identify the three data collection tools used for disease surveillance in Nigeria. This was similarly poor among the clinicians as only 7% of the clinicians could properly identify the three data collection tools (Table 2). Others had partial knowledge or no knowledge of the data collection tools. Some however, mentioned absolutely the data collection tools of the National Health Management Information System (NHMIS) which is managed as an independent data collection system from the disease surveillance system signifying some confusion.

**Table 1:** Distribution of private health facilities reporting notifiable diseases

Characteristics	Number of Facilities Surveyed	Proportion Reporting (%)
**State**		
Ekiti	20	35
Lagos	235	51
Ogun	118	17
Ondo	39	23
Osun	40	30
Oyo	55	60
Total (South-West)	507	40
**Type of Ownership**		
For Profit	445	41
Not for Profit	14	36
Unknown	48	31
**Facility Level**		
Primary	137	34
Secondary	135	54
Tertiary	5	45
Unknown	29	74

**Table 2:** Tool availability and behaviour

Questions	Categories	Responses	Percentage
**Does this facility have the tools for reporting notifiable diseases in Nigeria?**	**No**	334	66
	**Yes**	173	34
	**Total**	507	100
**Health facility has an assigned health records officer**	**No**	259	51
	**Yes**	248	49
	**Total**	507	100
**Health records officer assigned has training in health records/ information management**	**No**	167	67
	**Yes**	81	33
	**Total**	248	100
**Identification of three data collection tools for disease surveillance reporting by health records officer**	**Correct**	13	5
	**Partially Correct**	49	20
	**NHMIS**	27	11
	**Wrong**	159	64
	**Total**	248	100
**Identification of three data collection tools for disease surveillance reporting by Clinician**	**Correct**	33	7
	**Partially Correct**	69	14
	**NHMIS**	9	2
	**Wrong**	398	78
	**Total**	507	100

The knowledge of the healthcare providers on disease surveillance observed in the study was diverse as presented in Table 3. More than 50% of the clinicians interviewed were aware of a law or regulation that required compulsory reporting of notifiable diseases to the health authorities. About 76% of the clinicians scored at least 2 (out of a maximum of 3) in identifying three immediately notifiable diseases. However, most identified Polio, EVD and Lassa fever which are the main epidemic prone diseases that have recently ravaged the country and repeatedly echoed on the news. Thus, their identification of these three diseases may not be unconnected to the increased sensitization from the media. Only 228 (45%) of the health facilities have a link with, or the contact information for the LGA focal person for surveillance within their locality. Most facilities (85%) did not receive feedback or surveillance reports from the LGA authorities on disease outlook within their locality. Two-hundred and six (41%) of the clinician respondents noted that they had attended to a case that they believe should be reported to the health authorities. However, of these providers, only 106 (51%) succeeded in reporting to the health authorities. Of respondents that claimed not to have seen a case that needed to be reported to the authorities, over 90% of them had attended to at least one case of malaria within the year preceding the survey. Assessment of the performance of the disease surveillance system by private healthcare workers revealed that 39% of providers thought the system was either excellent or good while majority (61%) were either indifferent about the system, thought it was poor or non-existent. Findings from the regression analysis (Table 4) revealed that health facilities with clinicians aware of a law to report (OR=1.55 95% CI=1.08-2.24), had the requisite data collection tools (OR=13.69, 95% CI=8.85-21.62), had an assigned health records officer (OR=3.9, 95% CI=2.68-5.73), with health records personnel knowledgeable on the disease surveillance tools (OR=10.51, 95%CI=2.86-67.70) as well as had a clinician knowledgeable about the data collection tools (OR=2.49, 95% CI=1.22-5.25) were more likely to have complied with reporting into the disease surveillance system. However, the duration the health records officer had spent in the health facility, the length of time the physician (where available) had been out of medical school or whether the clinician agreed it was their responsibility to report did not have an effect on their participation in the disease surveillance system.

**Table 3:** Health worker knowledge and performance of disease surveillance system

Questions	Categories	Number of Respondents	Percentage
**Awareness of Law/ Regulation on compulsory reporting**	**No**	222	44
	**Yes**	285	56
	**Total**	507	100
**Score of clinician’s knowledges of immediately notifiable diseases**	**0**	46	9
	**1**	76	15
	**2**	153	30
	**3**	232	46
	**Total**	507	100
**Have link or Contact information to LGA representative in charge of disease surveillance in locality**	**No**	279	55
	**Yes**	228	45
	**Total**	507	100
**Do you receive report/ feedback on disease outbreaks in your locality?**	**No**	436	86
	**Yes**	71	14
	**Total**	507	100
**Have you ever attended to cases that you think should be reported to the health authorities?**	**No**	301	59
	**Yes**	206	41
	**Total**	507	100
**Among those that said they had attended to a case that they think should be reported, the number that succeeded**	**No**	100	49
	**Yes**	106	51
	**Total**	206	100
**Among those that said no, those that had attended to at least a case of Malaria within the last one year.**	**No**	21	7
	**Yes**	269	93
	**Total**	290	100
**Rating of the disease surveillance system**	**Excellent**	21	4
	**Good**	175	35
	**Indifferent**	97	19
	**Poor**	194	39
	**Non-existent**	12	2
	**Total**	499	99

**Table 4:** Relationship between reporting and various predictive variables

Factors	Odds Ratio	Lower Limit (95% Confidence Interval)	Upper Limit (95% Confidence Interval)
**Availability of reporting tools**	13.69	8.85	21.62
**Availability of an assigned Health Records Officer**	3.9	2.68	5.73
**Duration health records officer has spent in the health facility**	1.000	0.999	1.001
**Knowledge of the data collection tools used for reporting notifiable diseases by health records officers**	10.51	2.86	67.70
**Knowledge of data collection tools used for reporting notifiable diseases by the clinician in attendance**	2.49	1.22	5.25
**Number of years since graduation of the physician (where interviewed or responded).**	1.017	0.999	1.036
**Agree it is their responsibility to report**	1.88	0.40	3.91
**Awareness of a law to report**	1.55	1.08	2.24
**Level of Care Provided**			
Primary	Ref.	Ref.	Ref.
Secondary	1.67	1.14	2.45
Tertiary	2.35	0.68	8.41

## Discussion

Despite the significant contribution that private health facilities make to health service delivery across several low and middle income countries, their participation in disease surveillance and notification systems are generally poor [22]. The overall poor compliance with disease surveillance as seen in this study is in keeping with the findings reported by Phalkey and colleagues in their systematic review [22]. There was a large variation in compliance with the disease surveillance and notification system across states. This may be a reflection of the variation in engagement and enforcement of disease surveillance priorities by the state government authorities which oversee disease surveillance in the states. The relatively high performance recorded in Lagos state compared to the other states may be a fallout of action that followed the EVD outbreak of 2014 which spread to Nigeria [28]. The index case in that outbreak had presented in a private health facility in Lagos. However, the compliance with disease surveillance in Lagos while above mid-mark still requires further attention. Studies of the notifiable disease system in Taiwan found that physicians in urban areas were more likely to be aware of the surveillance system and complying with reporting notifiable diseases than their peers in less urban areas [29]. This explanation could also be responsible for the higher proportion of health facilities reporting in Lagos state which is the most urban of the six states surveyed. However, it is noteworthy that Oyo state did slightly better than Lagos overall.

In an earlier study from Nigeria, state government officers (state epidemiologists) noted that they were implementing the national IDSR policy [30]. However, none of the state officers interviewed in the study could provide any evidence that their state had adopted and domesticated the national policy for disease surveillance. The unavailability of state laws or policies might be a reflection of the attitude towards disease surveillance by the state ministries and could have manifested in the overall poor performance with compliance by the private health facilities in this study. Health facilities communicate with the LGA authorities on disease surveillance using predefined data collection tools. As such, it was unsurprising that health facilities that reported having the relevant data collection tools were more likely to have complied with reporting than their peers that did not have the appropriate tools. Likewise, health facilities with health workers that had more knowledge of the data collection tools were more likely to have complied with reporting. Knowledge of health workers on data collection tools could probably have been gained during routine completion of the forms. Also, those that interacted with the forms would have sought clarifications on areas they found difficult thereby improving their knowledge on the general disease surveillance system over time. Knowledge of a significant proportion of the health workers on the diseases tracked by the surveillance system was sub-optimal. Despite over half of them noting that they had not attended to a case that they believed should have been reported to the health authorities, an overwhelming proportion had attended to at least one case of malaria in the preceding year. Thus, it was obvious that they were unaware that malaria was one of the diseases tracked by the monthly IDSR003 form. Such patchy knowledge on the diseases tracked by the surveillance system can result in incomplete reporting and missed opportunities for the early detection of outbreaks. While the study made effort to ensure objectivity, there were some limitations in its execution. Information on compliance with reporting by the health facilities was based on self-reports by the health facility workers. As such, the number of facilities claiming to be submitting routine reports may have been overestimated since many of them were equally aware of the compulsory need for reporting and may have been afraid of the consequences of not doing so. Also, we found some of the respondents to have identified the National Health Management Information System tools as the disease surveillance data collection tools. As such, they equated the National Health Management Information System to the IDSR which is run as a parallel system. Thus, the proportion of health facilities noted to be reporting into the disease surveillance system may be much lower than has been reported in this study.

## Conclusion

This study on compliance with disease surveillance by private health facilities provides a quantitative picture of the suboptimal participation of private health facilities in the disease surveillance system in Nigeria. It also provides a view of the poor knowledge of health workers within them on the disease surveillance system. The findings are in keeping with earlier studies that revealed the poor participation of private health facilities in disease surveillance system in other low- and middle-income countries. Poor compliance with disease surveillance by private health facilities result in missed opportunities for the early warning system aimed at the prompt detection and response to infectious disease outbreaks. Efforts targeted at improving the performance of the disease surveillance system must include education of private health workers, the provision of data collection tools at the point of need and enforcements of international and national statutes on disease surveillance.

### What is known about this topic

- Routine disease surveillance serves as an important means of achieving the International Health Regulations;
- There is limited knowledge on the performance of private health facilities in disease surveillance systems in Nigeria.

### What this study adds

- This study provides a first level of analysis of the participation and performance of the private sector in the disease surveillance system in Nigeria;
- It provides a call to action to improve participation of private health facilities in the disease surveillance system as an early warning system for infectious disease outbreaks.

## Competing interests

The authors declare no competing interests.
